# Integrated measures for rough sets based on general binary relations

**DOI:** 10.1186/s40064-016-1670-2

**Published:** 2016-02-09

**Authors:** Shuhua Teng, Fan Liao, Mi He, Min Lu, Yongjian Nian

**Affiliations:** Science and Technology on Automatic Target Recognition Laboratory, National University of Defense Technology, Changsha, 410073 China; PLA Units: 66295, Baoding, 072750 China; School of Biomedical Engineering, Third Military Medical University, Chongqing, 400038 China

**Keywords:** Rough set, Uncertainty measure, General binary relation, Information system

## Abstract

Uncertainty measures are important for knowledge discovery and data mining. Rough set theory (RST) is an important tool for measuring and processing uncertain information. Although many RST-based methods for measuring system uncertainty have been investigated, the existing measures cannot adequately characterise the imprecision of a rough set. Moreover, these methods are suitable only for complete information systems, and it is difficult to generalise methods for complete information systems to incomplete information systems. To overcome these shortcomings, we present new uncertainty measures, integrated accuracy and integrated roughness, that are based on general binary relations, and we study important properties of these measures. A theoretical analysis and examples show that the proposed integrated measures are more precise than existing uncertainty measures, they are suitable for both complete and incomplete information systems, and they are logically consistent. Therefore, integrated accuracy and integrated roughness overcome the limitations of existing measures. This research not only develops the theory of uncertainty, it also expands the application domain of uncertainty measures and provides a theoretical basis for knowledge acquisition in information systems based on general binary relations.

## Background

Uncertainty is an important topic in research on artificial intelligence (Li and Du 2005). Rough set theory (RST) is a mathematical tool for handling imprecise, incomplete and uncertain data (Pawlak 1991), and it is an effective method to deal with uncertainty problems. In classical RST, the uncertainty of rough sets depends on two factors, knowledge uncertainty (the size of information granularities) and set uncertainty (the size of the rough set boundary) (Pawlak 1991). Set uncertainty in RST is measured with two quantities, accuracy and roughness, but they do not adequately reflect the uncertainty of a rough set. In some cases, the accuracy measure reflects only the size of the boundary region but not the size of the information granularities formed by the attribute sets, which limits the applicability of classical rough sets (Pawlak 1991). To solve this problem, researchers have proposed a number of integrated uncertainty measures based on certain binary relations (Teng et al. 2016; Wang et al. 2008a; Liang et al. 2009) that consider both the knowledge uncertainty and the set uncertainty. Although these measures are effective, they have certain restrictions. These measures change with information granularities which are unrelated to rough set *X*, i.e., information granularities in the negative region of *X*; this is inconsistent with human cognition in uncertainty problems (Wang and Zhang 2008). Intuitively, a rough measure that reflects two types of uncertainty should have a higher value than that of a measure which reflects only one type of uncertainty, but this property is not satisfied by the existing integrated uncertainty measures. In addition, the existing integrated uncertainty measures do not sufficiently characterise the uncertainty in certain cases. Wang and Zhang (2008) proposed a fuzziness measure for rough sets based on information entropy, which overcomes the problem of existing uncertainty measures for rough sets. However, a fuzziness measure based on the equivalence relation is not suitable for the incomplete information system and ordered information system. In practice, knowledge acquisition usually involves information that is incomplete for various reasons such as data measurement errors, a limited understanding and the conditions under which the data were acquired (Kryszkiewicz et al. 1998). Incompleteness in an information system is one of the main causes of uncertainty. RST, which is based on the traditional equivalence relation (i.e., reflexivity, symmetry, and transitivity) cannot directly deal with incomplete information systems, which greatly constrains the use of RST in practical applications (Gantayat et al. 2014; Sun et al. 2014). Hence, several extended models and methods for RST such as the tolerance relation (i.e., reflexivity, symmetry) (Wang and Zhang 2008), the asymmetric similarity relation (i.e., reflexivity, transitivity) (Stefanowski and Tsoukias 1999), the limited tolerance relation (i.e., reflexivity, symmetry) (Wang 2002), the dominance relation (reflexivity, transitivity) (Greco et al. 2002; Hu et al. 2012), and the general binary relation (i.e., reflexivity) (Yao 1998; Teng et al. 2009; Zhu 2007) which can directly process an incomplete information system, have been proposed. Based on these relations, directly measuring the uncertainty of incomplete data has caused considerable concern (Huang et al. 2004; Qian et al. 2009; Xu and Li 2011; Dai and Xu 2012; Sun et al. 2012; Dai et al. 2014; Chen et al. 2014; Dai et al. 2013).

The various uncertainty measures mentioned above are mostly aimed at one special binary relation without universality, and do not adequately reflect the uncertainty of rough sets in certain cases. Little attention has been paid to uncertainty measures based on general binary relations (Huang et al. 2004; Wang et al. 2008b). To overcome the limitations of the existing uncertainty measures and to analyse data more efficiently, it is necessary to find an uncertainty measure that is universal and more accurate.

This paper begins with an analysis of the limitations of the existing uncertainty measures for rough sets. Next, a knowledge uncertainty measure based on general binary relations is presented, which is applicable in classical systems as well, i.e., it is an effective technique to deal with complex data sets. Novel integrated measures based on general binary relations are proposed, and the properties of these integrated measures are analysed. At last, Examples are used to verify the validity of the proposed uncertainty measures.

## Preliminary concepts of RST

Information system is a pair *S* = (*U*, *A*), where $U = \{ u_{1} ,u_{2} , \ldots ,u_{\left| U \right|} \}$ is a non-empty finite set of objects (|∙| denotes the cardinality of the set), $A = \{ a_{1} ,a_{2} , \ldots ,a_{\left| A \right|} \}$ is a non-empty finite set of attributes such that $a_{j}$: $a_{j} \to V_{{a_{j} }}$ for every $a_{j} \in A$. The set $V_{{a_{j} }}$ is called the value set of $a_{j}$.

Each subset of attributes $P \subseteq A$ determines a binary indiscernibility relation $\text{IND(}P\text{)}$ as follows:

1 $$ {\text{IND}}(P) = \left\{ {\left( {u_{i} ,u_{j} } \right) \in U \times U\left| {\forall a \in P,f\left( {u_{i} ,a} \right) = } \right.f\left( {u_{j} ,a} \right)} \right\} $$

Obviously, $\text{IND(}P\text{)}$ is an equivalence relation. If $\left( {u_{i} ,u_{j} } \right) \in \text{IND(}P\text{)}$, then $u_{i}$ and $u_{j}$ are indiscernible with respect to attribute set *P*. The partition generated by $\text{IND(}P\text{)}$ is denoted by $U/{\text{IND}}(P)$, which can be abbreviated as $U/P$. The partition $U/P = \{ P_{1} ,P_{2} , \ldots ,P_{m} \}$ denotes knowledge associated with the equivalence relation $\text{IND(}P\text{)}$, where $P_{i}$ is an equivalence class, $1 \le i \le m$, and $1 \le m \le \left| U \right|$. Each equivalence class is an information granularity. Thus, the attribute set *P* will also be called the knowledge. The equivalence class determined by $u_{i}$ with respect to the attribute set *P* is denoted by $\left[ {u_{i} } \right]_{P} = \left\{ {u_{j} \in U|(u_{i} ,u_{j} ) \in \text{IND(}P\text{)}} \right\}$. Obviously, if $u_{i} \in P_{k}$, then $\left[ {u_{i} } \right]_{P} = P_{k}$. For any set $X \subseteq U$, the *P*-lower and *P*-upper approximations of *X* are $\underline{P} X = \{ u_{i} \in U|\left[ {u_{i} } \right]_{P} \subseteq X\}$ and $\overline{P} X = \{ u_{i} \in U|\left[ {u_{i} } \right]_{P} \cap X \ne \emptyset \}$, respectively. The boundary region of *X* is represented by $BN_{P} (X) = \overline{P} X - \underline{P} X$.

An information system *S* (= (*U*, *A*)) is an incomplete information system if the attribute values include an empty value “*”; otherwise, *S* is a complete information system.

In an information system, a relation derived from the attribute sets is generally not an equivalence relation but a general binary relation. In this paper, we use $R^{P}$ to represent a general binary relation derived from the knowledge *P*. In an information system *S*, $P \subseteq A$. We define the function $R_{S}^{P}$ as follows:

1. The set-valued function $R_{S}^{P} :U \to P(U)$ is defined as $R_{S}^{P} (u_{i} ) = \{ u_{j} \in U|(u_{i} ,u_{j} ) \in R^{P} \}$, where $R_{S}^{P} (u_{i} )$ is the subsequent neighbour of $u_{i}$ under the binary relation $R^{P}$. The relation $R^{P}$ and the corresponding subsequent neighbour $R_{S}^{P} (u_{i} )$ can be uniquely determined from each other, i.e., $u_{i} R^{P} u_{j} \Leftrightarrow u_{j} \in R_{S}^{P} (u_{i} )$. Let ${U \mathord{\left/ {\vphantom {U {R^{P} }}} \right. \kern-0pt} {R^{P} }} = \{ R_{S}^{P} (u_{i} )\left| {u_{i} \in U} \right.\}$ represent the classification of *U* divided by the knowledge *P*, where $R_{S}^{P} (u_{i} )$ is called a classification granularity under the general binary relation. The classification granularity $R_{S}^{P} (u_{i} )$ can be understood as the largest set of objects that cannot be distinguished from object $u_{i}$ given the knowledge *P*; i.e., objects in $R_{S}^{P} (u_{i} )$ should belong to the same class as $u_{i}$ given the knowledge *P*. Obviously, $R_{S}^{P} (u_{i} )$ will be an equivalence class, a dominance class, a tolerance class, a limited tolerance class, or an asymmetric similarity class of an object $u_{i}$ if $R^{P}$ is an equivalence relation, a dominance relation, a tolerance relation, a limited tolerance relation or an asymmetric similarity relation, respectively. Note that classification granularities in ${U \mathord{\left/ {\vphantom {U {R^{P} }}} \right. \kern-0pt} {R^{P} }}$ do not always constitute partitions or covers of *U* (Wang et al. 2008b). The lower and upper approximation sets of $X \subseteq U$ with respect to a general binary relation $R^{P}$ are defined as $\underline{{R^{P} }} (X) = \{ u_{i} \in U|R_{S}^{P} (u_{i} ) \subseteq X\}$ and $\overline{{R^{P} }} (X) = \{ u_{i} \in U|R_{S}^{P} (u_{i} ) \cap X \ne \emptyset \}$, respectively.
2. If $$ Q $$ and $$ P \subseteq A $$, we define a partial relation $$ \underset{\raise0.3em\hbox{$\smash{\scriptscriptstyle-}$}}{ \prec } $$ as follows: $$ P\underset{\raise0.3em\hbox{$\smash{\scriptscriptstyle-}$}}{ \prec } Q \Leftrightarrow R_{S}^{P} (u_{i} ) \subseteq R_{S}^{Q} (u_{i} ) $$ for $$ \forall u_{i} \in U $$, which means that the knowledge *P* is finer (i.e., has finer classification granularities) than the knowledge *Q*. If $$ R_{S}^{P} (u_{i} ) \subseteq R_{S}^{Q} (u_{i} ) $$ for $$ \forall u_{i} \in U $$ and $$ \exists u_{j} \in U $$ satisfies $$ R_{S}^{P} (u_{j} ) \subset R_{S}^{Q} (u_{j} ) $$, then we say that the knowledge *P* is strictly finer than the knowledge *Q*, or the knowledge *Q* entirely depends on the knowledge *P*, which is denoted by $$ P \prec Q $$. The notation $$ P \approx Q $$ represents $$ R_{S}^{P} (u_{i} ) = R_{S}^{Q} (u_{i} ) $$ for $$ \forall u_{i} \in U $$.

## Limitations of existing uncertainty measures

In classical RST, there are two main causes of uncertainty: the information granularity derived from the binary relation in the universe, which is knowledge uncertainty, and the boundary of the rough set in the given approximation space, which is set uncertainty (Pawlak 1991). Beaubouef et al. (1998) proposed a new integrated uncertainty measure for complete information systems, which they called rough entropy.

Given an information system $S = (U,A)$, $P,Q \subseteq A$, and $U/P = \{ P_{1} ,P_{1} , \ldots ,P_{m} \}$. The rough entropy of $X \subseteq U$ with respect to *P* is defined as (Beaubouef et al. 1998)

2 $$ H(X,P) = \rho_{P} (X)H^{G} (P) $$

where $$ {\kern 1pt} H^{G} (P) = - \left| {\sum\nolimits_{i = 1}^{m} {\frac{{\left| {P_{i} } \right|}}{\left| U \right|}\log_{2} \frac{1}{{\left| {P_{i} } \right|}}} } \right| $$ is called the granularity measure of the knowledge *P*. In Eq. (2), $$ H^{G} (P) $$ measures knowledge uncertainty, and the roughness $$ \rho_{P} (X) = 1 - \frac{{\left| {\underset{\raise0.3em\hbox{$\smash{\scriptscriptstyle-}$}}{P} X} \right|}}{{\left| {\bar{P}X} \right|}} $$ measures set uncertainty. Rough entropy considers two types of uncertainty and is therefore an integrated uncertainty measure.

Yang and John (2008) noted that existing uncertainty measures cannot correctly measure the uncertainty of boundary rough sets, whose lower approximation is an empty set. Thus, Yang and John (2008) defined the measures global accuracy $\sigma_{P} (X)$ and global roughness $G_{P} (X)$ under the equivalence relation to measure the uncertainty of rough sets:

3 $$ \sigma_{p} \left( X \right) = \frac{{\left| {U - BN_{P} \left( X \right)} \right|}}{\left| U \right|} $$

4 $$ G_{P} \left( X \right) = 1 - \sigma_{p} \left( X \right) $$

where $BN_{P} (X) = \left| {\overline{P} X} \right| - \left| {\underline{P} X} \right|$. The global accuracy and the global roughness reveal the global uncertainty with respect to the universe of discourse, which addresses the shortcomings of classical measures for boundary rough sets. However, similar to classical measures, global accuracy and global roughness cannot measure the knowledge uncertainty.

If the boundary region of $X \subseteq U$ with respect to the knowledge *A* is an empty set, the rough set *X* can be precisely described by the knowledge *A*. In this case, the rough set *X* becomes a precise set; i.e., the uncertainty of *X* is 0. Thus, the uncertainty of a rough set *X* is related only to the size of the boundary region and the information granularity of the boundary region and not to the information granularity in the positive and negative regions (Wang and Zhang 2008). Although the rough entropy in Eq. (2) can measure two types of uncertainty, it is not always effective in certain cases. In the following, two examples reveal the limitations of the existing uncertainty measures for both complete and incomplete information systems.

### *Example 1*

**Fig. 1:**
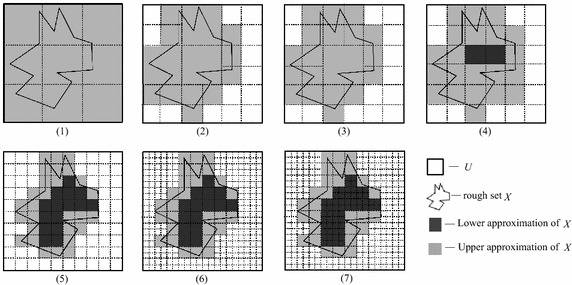
Lower and upper approximations of a rough set for various levels of information granularities

In a complete information system $S = (U,A)$, $U = \{ u_{1} ,u_{2} , \ldots ,u_{3600} \}$, $X \subseteq U$ and $P \subseteq A$. Figure 1 presents the lower and upper approximations and the boundary region of *X* as the information granularity induced by the knowledge *P* changes, where in subfigures (1)–(7) the information granularity is progressively finer. In subfigure (1), the lower approximation set is an empty set and the boundary region is the entire universe. Parts of the universe in Fig. 1 (2) are finer than those in Fig. 1 (1), i.e., 6 units in Fig. 1 (1) are equally divided into 24 smaller units. The lower approximation set remains empty, and the boundary region comprises 22 smaller units. Similarly, Fig. 1 (3) shows the results as parts of the universe [i.e., two of the large units in Fig. 1 (2)] are further divided evenly. Figure 1 (4) presents the results when the largest unit in Fig. 1 (3) is further divided evenly. Figure 1 (5) shows the results when all of the smaller units in Fig. 1 (4) are further divided evenly, and Fig. 1 (6) presents the results when the negative region of Fig. 1 (5) is divided evenly. Figure 1 (7) presents the results when the positive domain of Fig. 1 (6) is further divided evenly.

The values of various uncertainty measures for the rough set *X* in each subfigure of Fig. 1 are shown in Table 1, where *Num_L*, *Num_U* and *Num_B* represent the number of objects in the lower approximation, the upper approximation, and the boundary region, respectively. From Table 1, we can observe that the number of objects in the boundary of *X* decreases as the information granularity becomes finer, i.e., the number of objects surely belonging or not belonging to *X* increases. The uncertainty measures decrease monotonically as the information granularities become smaller through finer classification. However, the existing uncertainty measures are not always effective in certain cases; their limitations are revealed by the following five observations:

**Table 1:** Uncertainty measures of the rough set *X* with various information granularities

Subfig. no.	Uncertainty measures						
	*Num_L*	*Num_U*	*Num_B*	$\rho_{P} (X)$	$G_{P} (X)$	$H^{G} (P)$	$H(X,P)$
(1)	0	3600	3600	1	1	8.64	8.64
(2)	0	2200	2200	1	0.61	7.31	7.31
(3)	0	2200	2200	1	0.61	6.87	6.87
(4)	200	2200	2000	0.91	0.56	6.64	6.04
(5)	5400	1548	1008	0.65	0.28	5.17	3.36
(6)	5400	1548	1008	0.65	0.28	4.03	2.62
(7)	5400	1548	1008	0.65	0.28	3.25	2.11

1. Rough set *X* is a boundary rough set (i.e., the lower approximation of *X* is an empty set) in Figs. 1 and 2. From the differences between partitions (1) and (2), we can observe that the boundary region becomes smaller and the information granularities in the boundary region become finer. Obviously, the uncertainty of the rough set *X* should become smaller. However, $\rho_{P} (X)$ in Table 1 does not change; although $H^{G} (P)$ decreases, it reflects only the variation in the information granularity and not the uncertainty of the set. Thus, $\rho_{P} (X)$ and $H^{G} (P)$ cannot adequately describe the uncertainty of a boundary rough set. The measure $H(X,P)$ reflects only the set uncertainty of the boundary rough set and not the knowledge uncertainty.

**Fig. 2:**
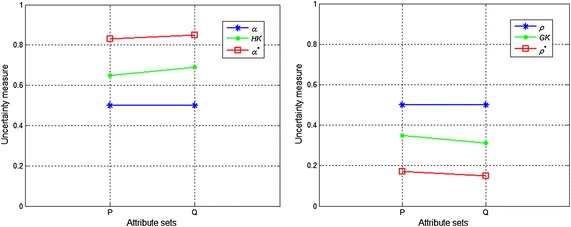
Uncertainty measures when *X* = *X*_1_
2. It can be observed that from partitions (2) and (3) that the boundary region does not change, but the information granularity in the boundary region becomes finer, which shows that the set uncertainty remains the same while the knowledge uncertainty decreases. In Table 1, $\rho_{P} (X)$ and $G_{P} (X)$ do not change whereas $H(X,P)$ decreases, which illustrates that $\rho_{P} (X)$ and $G_{P} (X)$ do not reflect the uncertainty of the knowledge whereas rough entropy $H(X,P)$ does.
3. Comparing partitions (3) with (4) and (4) with (5), it can be observed that the boundary region becomes smaller and the information granularity in the boundary region becomes finer. Therefore, the uncertainty of the rough set *X* decreases. In Table 1, $\rho_{P} (X)$, $G_{P} (X)$, $H^{G} (P)$ and $H(X,P)$ all decrease. However, $\rho_{P} (X)$ and $G_{P} (X)$ reflect only the set uncertainty, $H^{G} (P)$ reflects only the knowledge uncertainty, and $H(X,P)$ reflects both types of uncertainty.
4. Comparing partitions (5) with (6) and (6) with (7), we can observe that the boundary region and the information granularity in the boundary region remain the same. Accordingly, the uncertainty of *X* should not change (Wang and Zhang 2008). Although the information granularity becomes finer in the negative region from (5) to (6) and in the positive region from (6) to (7), the uncertainty of rough set *X* should remain unaffected (Wang and Zhang 2008). In Table 1, $\rho_{P} (X)$ and $G_{P} (X)$ are constant, which is consistent with human cognition, but $H(X,P)$ decreases, which shows that $H(X,P)$ does not accurately reflect the uncertainty of a rough set in this case.
5. An integrated measure of uncertainty in RST includes both types of uncertainty. Intuitively, the value of an integrated roughness measure that includes both types of uncertainty should be larger than that of a measure that considers only one type of uncertainty. However, rough entropy does not satisfy this requirement: although rough entropy includes both types of uncertainty, the numerical values can be smaller than those of the knowledge uncertainty measure, as shown in Table 1.

From the preceding analysis, it may be concluded that the existing uncertainty measures for a complete information system do not accurately reflect the uncertainty of rough sets. Next, the characteristics of uncertainty measures for an incomplete information system will be analysed.

In an incomplete information system, the equivalence relation of classical measures is extended to a tolerance relation $R_{T}^{P}$, which is expressed as:

5 $$ \alpha_{{R_{{_{T} }}^{P} }} (X) = \frac{{\left| {\underline{{R_{T}^{P} }} (X)} \right|}}{{\left| {\overline{{R_{T}^{P} }} (X)} \right|}} $$

6 $$ \rho_{{R_{{_{T} }}^{P} }} (X) = 1 - \alpha_{{R_{{_{T} }}^{P} }} (X) $$

In Eqs. (5) and (6), $\alpha_{{R_{{_{T} }}^{P} }} (X)$ and $\rho_{{R_{{_{T} }}^{P} }} (X)$ are the accuracy and the roughness, respectively. Obviously, $0 \le \alpha_{{R_{{_{T} }}^{P} }} (X),\rho_{{R_{{_{T} }}^{P} }} (X) \le 1$. The larger the uncertainty of a rough set, the smaller $\alpha_{{R_{{_{T} }}^{P} }} (X)$ is and the larger $\rho_{{R_{{_{T} }}^{P} }} (X)$ is. Therefore, the accuracy and the roughness can be used to measure the set uncertainty. As was the case for a complete information system, Eqs. (5) and (6) measure only set uncertainty and not knowledge uncertainty for an incomplete information system (Wang et al. 2008a). Wang et al. (2008a) proposed new definitions of accuracy and roughness based on the tolerance relation:

7 $$ \alpha_{{R_{{_{T} }}^{P} }}^{*} (X) = 1 - \rho_{{R_{{_{T} }}^{P} }} (X) \times GK(R_{{_{T} }}^{P} ) $$

8 $$ \rho_{{R_{{_{T} }}^{P} }}^{*} (X) = \rho_{{R_{{_{T} }}^{P} }} (X) \times GK(R_{{_{T} }}^{P} ) $$

Knowledge granularity, defined as $GK(R_{{_{T} }}^{P} ) = \sum\nolimits_{i = 1}^{\left| U \right|} {{{\left| {R_{{_{T} }}^{P} (u_{i} )} \right|} \mathord{\left/ {\vphantom {{\left| {R_{{_{T} }}^{P} (u_{i} )} \right|} {\left| U \right|^{2} }}} \right. \kern-0pt} {\left| U \right|^{2} }}}$, was employed to measure the roughness of knowledge. In contrast to knowledge granularity, $HK(R_{{_{T} }}^{P} ) = 1 - GK(R_{{_{T} }}^{P} )$ was used to characterise the precision of knowledge. Obviously, Eqs. (7) and (8) consider both set uncertainty and knowledge uncertainty, which corrects the problems with the classical definitions of accuracy and roughness to some extent. However, certain limitations remain for an incomplete information system, and these are revealed by the following example.

### *Example 2*

Let $S = (U,A)$ be an incomplete information system with $U = \{ u_{1} ,u_{2} , \ldots ,u_{7} \}$, $P,Q \subseteq A$. Assume that

9 $$ \left\{ {\begin{array}{l} {{U \mathord{\left/ {\vphantom {U {R_{{_{T} }}^{P} }}} \right. \kern-0pt} {R_{{_{T} }}^{P} }} = \left\{ {\{ u_{1} ,u_{2} \} ,\{ u_{2} ,u_{1} \} ,\{ u_{3} ,u_{4} ,u_{5} \} ,\{ u_{4} ,u_{3} \} ,\{ u_{5} ,u_{3} ,u_{6} \} ,\{ u_{6} ,u_{5} ,u_{7} \} ,\{ u_{7} ,u_{6} \} } \right\}} \\ {{U \mathord{\left/ {\vphantom {U {R_{{_{T} }}^{Q} }}} \right. \kern-0pt} {R_{{_{T} }}^{Q} }} = \left\{ {\{ u_{1} ,u_{2} \} ,\{ u_{2} ,u_{1} \} ,\{ u_{3} ,u_{4} \} ,\{ u_{4} ,u_{3} \} ,\{ u_{5} ,u_{6} \} ,\{ u_{6} ,u_{5} ,u_{7} \} ,\{ u_{7} ,u_{6} \} } \right\}} \\ \end{array} } \right. $$

**Fig. 3:**
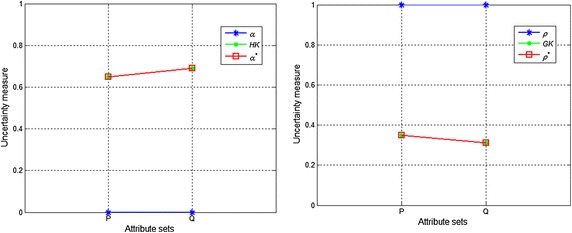
Uncertainty measures when *X* = *X*_2_

Obviously, ${U \mathord{\left/ {\vphantom {U {R_{{_{T} }}^{Q} }}} \right. \kern-0pt} {R_{{_{T} }}^{Q} }} \subset {U \mathord{\left/ {\vphantom {U {R_{{_{T} }}^{P} }}} \right. \kern-0pt} {R_{{_{T} }}^{P} }}$. Table 2 shows the upper and lower approximations, and the boundary region of the rough set *X*, while Table 3 shows the values of the uncertainty measures of the rough sets *X* for the knowledge *P* and *Q*. Figures 2 and 3 present the uncertainty measures of $X_{1}$ and $X_{2}$, respectively. The subscripts of the uncertainty measures in Figs. 2 and 3 are omitted, e.g., $\alpha_{{R_{{_{T} }}^{P} }} (X)$ is abbreviated as $\alpha$ and $GK(R_{{_{T} }}^{P} )$ is abbreviated as $GK$.

**Table 2:** Upper and lower approximations and the boundary region of the rough set *X*

*X*	$\underline{{R_{T}^{P} }} (X)$	$\underline{{R_{T}^{Q} }} (X)$	$\overline{{R_{T}^{P} }} (X)$	$\overline{{R_{T}^{Q} }} (X)$	$BN_{{R_{T}^{P} }} (X)$	$BN_{{R_{T}^{Q} }} (X)$
*X*_1_	{*u*_1_, *u*_2_}	{*u*_1_, *u*_2_}	{*u*_1_, *u*_2_, *u*_6_, *u*_7_}	{*u*_1_, *u*_2_, *u*_6_, *u*_7_}	{*u*_6_, *u*_7_}	{*u*_6_, *u*_7_}
*X*_2_	$\emptyset$	$\emptyset$	{*u*_1_, *u*_2_, *u*_3_, *u*_4_, *u*_5_}	{*u*_1_, *u*_2_, *u*_3_, *u*_4_}	{*u*_1_, *u*_2_, *u*_3_, *u*_4_, *u*_5_}	{*u*_1_, *u*_2_, *u*_3_, *u*_4_}

**Table 3:** Uncertainty measures of the rough set *X*

*X*	$\rho_{{R_{T}^{P} }} (X)$	$\rho_{{R_{T}^{Q} }} (X)$	$GK(R_{T}^{P} )$	$GK(R_{T}^{Q} )$	$\rho_{{R_{{_{T} }}^{P} }}^{*} (X)$	$\rho_{{R_{{_{T} }}^{Q} }}^{*} (X)$
*X*_1_	0.50	0.50	0.35	0.31	0.17	0.15
*X*_2_	1	1	0.35	0.31	0.35	0.31

From Tables 2 and 3, Figs. 2, and 3, we can make the following observations:

1. When $X = X_{1}$, the lower and upper approximations of $X_{1}$ with respect to the knowledge *P* and *Q* are identical, and the classification granularities in the upper approximations {*u*_1_, *u*_2_, *u*_6_, *u*_7_} induced by the knowledge *P* and *Q* are also identical. Therefore, the roughness and the accuracy of the knowledge *P* and *Q* are equal, which is logically consistent. However, $\alpha_{{R_{{_{T} }}^{P} }}^{*} (X) < \alpha_{{R_{{_{T} }}^{Q} }}^{*} (X)$ and $\rho_{{R_{{_{T} }}^{Q} }}^{*} (X) < \rho_{{R_{{_{T} }}^{P} }}^{*} (X)$. These results are caused by the subdivision of the classification granularities $R_{{_{T} }}^{P} (u_{3} )$ and $R_{{_{T} }}^{P} (u_{5} )$ in the negative region of set $X_{1}$ with the knowledge *Q*. Obviously, $R_{{_{T} }}^{P} (u_{3} )$ and $R_{{_{T} }}^{P} (u_{5} )$ are unrelated to *X*, and thus $\alpha_{{R_{{_{T} }}^{P} }}^{*} (X)$ and $\rho_{{R_{{_{T} }}^{P} }}^{*} (X)$ are inconsistent with human cognition.
2. When $X = X_{2}$, the lower approximation of set $X_{2}$ is an empty set, and as a result, $X_{2}$ is a boundary rough set. The boundary regions of $X_{2}$ with respect to the knowledge *P* and *Q* are different. In this case, the larger the boundary region is, the coarser the knowledge (Yang and John 2008). However, $\rho_{{R_{{_{T} }}^{P} }} (X) = \rho_{{R_{{_{T} }}^{Q} }} (X)$ and $\alpha_{{R_{{_{T} }}^{P} }} (X) = \alpha_{{R_{{_{T} }}^{Q} }} (X)$, so from Tables 2 and 3 we obtain $HK(R_{{_{T} }}^{P} ) = \alpha_{{R_{{_{T} }}^{P} }}^{*} (X) < \alpha_{{R_{{_{T} }}^{Q} }}^{*} (X) = HK(R_{{_{T} }}^{Q} )$ and $GK(R_{{_{T} }}^{Q} ) = \rho_{{R_{{_{T} }}^{Q} }}^{*} (X) < \rho_{{R_{{_{T} }}^{P} }}^{*} (X) = GK(R_{{_{T} }}^{P} )$, which shows that $\rho_{{R_{{_{T} }}^{P} }} (X)$ and $\alpha_{{R_{{_{T} }}^{P} }} (X)$ do not accurately reflect the uncertainty of the rough set when $BN_{{R_{T}^{P} }} (X) = \emptyset$; $\alpha_{{R_{{_{T} }}^{P} }}^{*} (X)$ and $\rho_{{R_{{_{T} }}^{P} }}^{*} (X)$ can measure the knowledge uncertainty but not the set uncertainty.
3. From Fig. 2 and Fig. 3, it can be observed that $\alpha_{{R_{{_{T} }}^{P} }} (X) \le \alpha_{{R_{{_{T} }}^{P} }}^{*} (X)$, $\alpha_{{R_{{_{T} }}^{Q} }} (X) \le \alpha_{{R_{{_{T} }}^{Q} }}^{*} (X)$, $HK(R_{{_{T} }}^{P} ) \le \alpha_{{R_{{_{T} }}^{P} }}^{*} (X)$, and $HK(R_{{_{T} }}^{Q} ) \le \alpha_{{R_{{_{T} }}^{Q} }}^{*} (X)$; therefore, $\rho_{{R_{{_{T} }}^{P} }}^{*} (X) < \rho_{{R_{{_{T} }}^{P} }} (X)$, $\rho_{{R_{{_{T} }}^{Q} }}^{*} (X) < \rho_{{R_{{_{T} }}^{Q} }} (X)$, $\rho_{{R_{{_{T} }}^{P} }}^{*} (X) \le GK(R_{{_{T} }}^{P} )$ and $\rho_{{R_{{_{T} }}^{Q} }}^{*} (X) \le GK(R_{{_{T} }}^{Q} )$ when $X = X_{1}$ or $X = X_{2}$. That is, the value of the roughness measure that includes two types of uncertainty is smaller than that of the measure reflecting only one type of uncertainty, whereas the value of the accuracy measure that includes two types of uncertainty is greater than that of the measure reflecting only one type of uncertainty. Obviously, these results are logically inconsistent.

Example 2 shows that, similar to the results for a complete information system, uncertainty measures for an incomplete information system have certain limitations. Xu et al. (Xu et al. 2009) presented a new integrated uncertainty measure for ordered information systems with properties similar to those of $\alpha_{{R_{{_{T} }}^{P} }}^{*} (X)$ and $\rho_{{R_{{_{T} }}^{P} }}^{*} (X)$. Therefore, this uncertainty measure has the same limitations.

From Examples 1 and 2, we can conclude that the imprecision of rough sets is not well characterised by existing measures for both complete and incomplete information systems. Therefore, it is necessary to find a more comprehensive and effective uncertainty measure based on general binary relations.

## Integrated measures based on general binary relations

In classical RST (Pawlak 1991), uncertainty includes knowledge uncertainty and set uncertainty. Various integrated uncertainty measures have been proposed that are based on a given binary relation and include both types of uncertainty (Wang et al. 2008a; Liang et al. 2009; Xu et al. 2009). The values of these measures depend on the classification granularity, which is unassociated with the set $X \subseteq U$, specifically the classification granularity in the negative region of *X*. This behaviour is inconsistent with human cognition (Wang and Zhang 2008). Intuitively, the value of an integrated roughness measure (i.e., the roughness of a rough set) that evaluates two types of uncertainty should be greater than that of a measure which evaluates only one type of uncertainty, but this property is not satisfied by almost all the existing integrated measures. In addition, the existing integrated uncertainty measures cannot be used to effectively characterise the roughness of rough sets in certain cases. In this section, the limitations of existing integrated uncertainty measures are addressed. First, a knowledge uncertainty measure that is based on general binary relations is presented. Based on this uncertainty measure, novel and logically consistent integrated uncertainty measures are presented.

### Information entropy measure based on general binary relations

Classical RST starts from an equivalence relation. Knowledge is based on the ability to partition a “universe” using the equivalence relation. The finer the partitioning, the more precise the knowledge will be. In an incomplete information system, overlaps may occur among several similar classes defined by the tolerance relation, the similarity relation, or the limited tolerance relation. Moreover, a covering is substituted for the partition of the universe. Thus, the equivalence relation cannot be satisfied for an incomplete information system. The same problems appear for general binary relations. However, research on uncertainty measures based on general binary relations is lacking (Huang et al. 2004). This lack of research motivates the investigation of an effective uncertainty measure based on general binary relations. In the following, an uncertainty measure based on general binary relations will be discussed.

Let $R^{P} \subseteq U \times U$ be a general binary relation on *U*, $P \subseteq A$. For two elements $u_{i} ,u_{j} \in U$, if $u_{j}$ has the same properties as $u_{i}$ with respect to *R*^*P*^, i.e., $u_{i} R^{P} u_{j}$, we say that $u_{j}$ is $R^{P}$-related to $u_{i}$. A general binary relation may be more conveniently represented using successor neighbourhoods or a classification granularity:

10 $$ R_{S}^{P} (u_{i} ) = \{ u_{j} \left| {u_{j} \in U,u_{i} R_{S}^{P} u_{j} } \right.\} $$

The classification granularity $R_{S}^{P} (u_{i} )$ consists of all $R^{P}$-related elements of $u_{i}$. If $R_{S}^{P} (u_{i} )$ contains more elements, more objects will belong to the same class as $u_{i}$, the classification granularities will be larger, and the capability of the knowledge *P* to classify the object $u_{i}$ will be weaker. Given these characteristics, a definition of an uncertainty measure based on general binary relations is given as follows.

#### **Definition 1**

Given an information system $S = (U,A)$, $u_{i} \in U$ and $1 \le i \le \left| U \right|$, the information entropy of the knowledge $P \subseteq A$ is defined as

11 $$ H^{\prime}(P) = 1 - G^{\prime}(P) $$

where

12 $$ G^{\prime}(P) = \sum\limits_{{u_{i} \in BN_{{R^{P} }} (X)}} {\frac{{\left| {R_{S}^{P} (u_{i} )} \right| - 1}}{\left| U \right|(\left| U \right| - 1)}} $$

#### **Theorem 1**

(Monotonicity) *Given an information system*$$ S = (U,A) $$, $$ P,Q \subseteq A $$*and*$$ P\underset{\raise0.3em\hbox{$\smash{\scriptscriptstyle-}$}}{ \prec } Q $$*, the information entropy satisfies*$$ H^{\prime}(Q) \le H^{\prime}(P) $$*, where equality holds if and only if*$$ P \approx Q $$.

The proof of this theorem follows from the definition of the partial relation and Definition 1.

#### **Corollary 1**

*Given an information system*$S = (U,A)$, $P \subseteq A$, $H^{{\prime }} (P)$*reaches a minimum value of 0 if and only if*$R_{S}^{P} (u_{i} ) = U$*for*$\forall u_{i} \in U$*, and*$H^{{\prime }} (P)$*reaches a maximum value of 1 if and only if*$R_{S}^{P} (u_{i} ) = u_{i}$*for*$\forall u_{i} \in U$.

Theorem 1 and Corollary 1 indicate that the information entropy monotonically increases as the classification granularity becomes smaller through finer classification. If the knowledge *P* cannot distinguish between any two objects in the universe *U*, the information entropy is at the minimum and the knowledge *P* has the weakest classification capability and the greatest roughness. If the knowledge *P* can distinguish all objects in the universe *U*, the information entropy is at the maximum and the knowledge *P* has the strongest classification capability and accuracy. Therefore, information entropy describes the roughness of knowledge in the context of granularity.

### Integrated measures of rough sets

To measure the uncertainty of rough sets more precisely, Yang and John (2008) proposed two complementary uncertainty measures for a complete information system, global accuracy and global roughness. These two complementary uncertainty measures can measure the set uncertainty more comprehensively than other uncertainty measures. However, these two complementary uncertainty measures are based on the equivalence relation and are not suitable for an incomplete information system. However, global accuracy and global roughness can be extended to incomplete systems using a general binary relation. The new definition for global accuracy is

13 $$ \sigma_{P}^{{\prime }} (X) = 1 - \frac{{\left| {BN_{P}^{{\prime }} (X)} \right|}}{2\left| U \right|} $$

where $BN_{P}^{{\prime }} (X) = \overline{{R^{P} }} (X) - \underline{{R^{P} }} (X)$. Global roughness is then defined as $\omega_{P}^{{\prime }} (X) = 1 - \sigma_{P}^{{\prime }} (X)$. Based on these definitions, we propose two novel integrated measures.

#### **Definition 2**

Given an information system $S = (U,A)$, $P \subseteq A$, $X \subseteq U$ and the general binary relation $R^{P}$, the integrated roughness and the integrated accuracy of *X* are defined as:

14 $$ \rho_{P}^{{\prime }} (X) = 1 - \alpha_{P}^{{\prime }} (X) $$

15 $$ \alpha_{P}^{{\prime }} (X) = \sigma_{P}^{{\prime }} (X) \times H^{{\prime }} (P) $$

$H^{{\prime }} (P)$ is used to measure knowledge uncertainty, and $\sigma_{P}^{{\prime }} (X)$ is used to measure set uncertainty. Obviously, Definition 2 considers not only the size of the boundary region of a rough set but also the classification granularity of the boundary region. Therefore, integrated roughness and integrated accuracy measure two types of uncertainty.

#### **Theorem 2**

(Monotonicity) *Given an information system*$S = (U,A)$, $P,Q \subseteq A$, $P \prec Q$*, and*$X \subseteq U$*, the following relations hold:*

(1) $$ \sigma^{\prime}_{Q} (X) \le \sigma^{\prime}_{P} (X) $$; (2) $$ \rho^{\prime}_{P} (X) \le \rho^{\prime}_{Q} (X) $$.

#### *Proof*

(1) Because $P \prec Q$, we have that $R_{S}^{P} (u_{i} ) \subseteq R_{S}^{Q} (u_{i} )$ for $\forall u_{i} \in U$, and $\exists u_{k} \in U$ satisfies $R_{S}^{P} (u_{k} ) \subset R_{S}^{Q} (u_{k} )$. For $\forall u_{i} \in \underline{{R^{Q} }} (X)$, $R_{S}^{Q} (u_{i} ) \subseteq X$, we obtain $R_{S}^{P} (u_{i} ) \subseteq X$, i.e., $u_{i} \in \underline{{R^{P} }} (X)$. Thus, $\underline{{R^{Q} }} (X) \subseteq \underline{{R^{P} }} (X)$. Similarly, $R_{S}^{P} (u_{i} ) \cap X \ne \emptyset$ for $\forall u_{i} \in \overline{{R^{P} }} (X)$. Because $R_{S}^{P} (u_{i} ) \subseteq R_{S}^{Q} (u_{i} )$, we have $R_{S}^{Q} (u_{i} ) \cap X \ne \emptyset$, i.e., $u_{i} \in \overline{{R^{Q} }} (X)$. Therefore, $\overline{{R^{P} }} (X) \subseteq \overline{{R^{Q} }} (X)$ and $BN^{\prime}_{P} (X) \subseteq BN^{\prime}_{Q} (X)$. According to Eq. (13), we have $\sigma_{Q}^{{\prime }} (X) \le \sigma_{P}^{{\prime }} (X)$, where equality holds if and only if $BN_{P}^{{\prime }} (X) = BN_{Q}^{{\prime }} (X)$.

(2) Because $P \prec Q$, we have $R_{S}^{P} (u_{i} ) \subseteq R_{S}^{Q} (u_{i} )$ for any $u_{i} \in U$, and $\exists u_{k} \in U$ satisfies $R_{S}^{P} (u_{k} ) \subset R_{S}^{Q} (u_{k} )$. To simplify the proof, we assume that only one object $u_{k} \in U$ satisfies $R_{S}^{P} (u_{k} ) \subset R_{S}^{Q} (u_{k} )$, so we have $R_{S}^{P} (u_{i} ) = R_{S}^{Q} (u_{i} )$ for any other $u_{i} \ne u_{k}$ (the proof for many objects is similar). Three cases are discussed:

① $R_{S}^{Q} (u_{k} ) \subseteq X$: Because $R_{S}^{P} (u_{k} ) \subset R_{S}^{Q} (u_{k} )$, it follows that $R_{S}^{P} (u_{k} ) \subseteq X$ and $u_{k} \notin BN_{P}^{{\prime }} (X) = BN_{Q}^{{\prime }} (X)$. From the proof of (1), we have $\sigma_{Q}^{{\prime }} (X) = \sigma_{P}^{{\prime }} (X)$. Because ${\kern 1pt} {\kern 1pt} R_{S}^{P} (u_{i} ) = R_{S}^{Q} (u_{i} )$ for $\forall u_{i} \ne u_{k}$, from Eq. (8) we obtain $H^{{\prime }} (P) = H^{{\prime }} (Q)$. According to Definition 2, we have $\alpha_{Q}^{{\prime }} (X) = \alpha_{P}^{{\prime }} (X)$ and $\rho_{P}^{{\prime }} (X) = \rho_{Q}^{{\prime }} (X)$.
② $R_{S}^{Q} (u_{k} ) \cap X = \emptyset$: Because $R_{S}^{P} (u_{k} ) \subset R_{S}^{Q} (u_{k} )$, we have $R_{S}^{P} (u_{k} ) \cap X = \emptyset$ and $u_{k} \notin BN_{P}^{{\prime }} (X) = BN_{Q}^{{\prime }} (X)$. From the proof of (1), we have $\sigma_{Q}^{{\prime }} (X) = \sigma_{P}^{{\prime }} (X)$. Because ${\kern 1pt} {\kern 1pt} R_{S}^{P} (u_{i} ) = R_{S}^{Q} (u_{i} )$ for $\forall u_{i} \ne u_{k}$, from Eq. (11) we obtain $H^{{\prime }} (Q) = H^{{\prime }} (P)$. According to Definition 2, we have that $\alpha_{Q}^{{\prime }} (X) = \alpha_{P}^{{\prime }} (X)$ and $\rho_{P}^{{\prime }} (X) = \rho_{Q}^{{\prime }} (X)$.
③ $R_{S}^{Q} (u_{k} ) \cap X \ne \emptyset$ and $R_{S}^{Q} (u_{k} ) \cap X \ne R_{S}^{Q} (u_{k} )$. We have $u_{k} \in BN_{Q}^{{\prime }} (X)$. Three cases must be considered:
  1. If $R_{S}^{P} (u_{k} ) \cap X \ne \emptyset$ and $R_{S}^{P} (u_{k} ) \cap X \ne R_{S}^{P} (u_{k} )$, then $u_{k} \in BN_{P}^{{\prime }} (X) = BN_{Q}^{{\prime }} (X)$. From the proof of (1), we obtain $0 < \sigma_{Q}^{{\prime }} (X) = \sigma_{P}^{{\prime }} (X)$. Because $R_{S}^{P} (u_{i} ) = R_{S}^{Q} (u_{i} )$ for $\forall u_{i} \ne u_{k}$, $R_{S}^{P} (u_{k} ) \subset R_{S}^{Q} (u_{k} )$, from Eq. (8) and Definition 2 we have that $H^{{\prime }} (Q) < H^{{\prime }} (P)$, $\alpha_{Q}^{{\prime }} (X) < \alpha_{P}^{{\prime }} (X)$ and $\rho_{P}^{{\prime }} (X) < \rho_{Q}^{{\prime }} (X)$.
  2. If $R_{S}^{P} (u_{k} ) \subseteq X$, then $u_{k} \notin BN_{P}^{{\prime }} (X)$. Thus, $BN_{P}^{{\prime }} (X) \subset BN_{Q}^{{\prime }} (X) \ne \emptyset$. From the proof of (1), we have $\sigma_{Q}^{{\prime }} (X) < \sigma_{P}^{{\prime }} (X)$. Because $R_{S}^{P} (u_{i} ) = R_{S}^{Q} (u_{i} )$ and $R_{S}^{P} (u_{k} ) \subset R_{S}^{Q} (u_{k} )$ for $\forall u_{i} \ne u_{k}$, according to Eq. (8) we have that $H^{{\prime }} (Q) < H^{{\prime }} (P)$. From Definition 2, we have that $\alpha_{Q}^{{\prime }} (X) < \alpha_{P}^{{\prime }} (X)$ and $\rho_{P}^{{\prime }} (X) < \rho_{Q}^{{\prime }} (X)$.
  3. If $R_{S}^{P} (u_{k} ) \cap X = \emptyset$, then $u_{k} \notin BN_{P}^{{\prime }} (X)$. Therefore, $BN_{P}^{{\prime }} (X) \subset BN_{Q}^{{\prime }} (X) \ne \emptyset$. From the proof of (1), we have $\sigma_{Q}^{{\prime }} (X) < \sigma_{P}^{{\prime }} (X)$. Because $R_{S}^{P} (u_{i} ) = R_{S}^{Q} (u_{i} )$ and $R_{S}^{P} (u_{k} ) \subset R_{S}^{Q} (u_{k} )$ for $\forall u_{i} \ne u_{k}$, according to Eq. (13) we obtain $H^{\prime}(Q) < H^{\prime}(P)$. From Definition 2, we have that $\alpha_{Q}^{{\prime }} (X) < \alpha_{P}^{{\prime }} (X)$ and $\rho_{P}^{{\prime }} (X) < \rho_{Q}^{{\prime }} (X)$.

This concludes the proof of Theorem 2.

#### **Corollary 2**

*Given an information system*$S = (U,A)$, $P,Q \subseteq A$, $P \prec Q$*and*$X \subseteq U$*, where*$U^{\prime} = \{ u_{k} \in U\left| {R_{S}^{P} (u_{k} ) \subset R_{S}^{Q} (u_{k} )} \right.\}$*, then*$\rho_{P}^{\alpha } (X) = \rho_{Q}^{\alpha } (X)$*if and only if*$u_{k} \notin BN_{Q}^{{\prime }} (X)$*for*$\forall u_{k} \in U^{{\prime }}$.

The proof of this corollary follows from Theorem 2. From Theorem 2 and Corollary 2, we can observe that the integrated accuracy does not strictly monotonically increase, and the integrated roughness does not strictly monotonically decrease as the classification granularity becomes smaller through finer classification. That is, the integrated accuracy and the integrated roughness are unrelated to the classification granularity $R_{S}^{Q} (u_{i} )$, where $u_{i} \in \{ U - BN^{\prime}_{Q} (X)\}$. If the classification granularity $R_{S}^{Q} (u_{k} )$ defined by the knowledge *P* satisfies $u_{k} \in BN^{\prime}_{Q} (X)$, the integrated accuracy (integrated roughness) strictly monotonically increases (decreases), which is accords to human cognition.

#### **Corollary 3**

*Given an information system*$S = (U,A)$, $P \subseteq A{\kern 1pt}$*and*$X \subseteq U$*, the integrated roughness satisfies*$0 \le \rho_{P}^{{\prime }} (X) \le 1$*. Equality holds on the right side if and only if*$R_{S}^{P} (u_{i} ) = U$*for*$\forall u_{i} \in U$*, and equality holds on the left side if and only if*$BN_{P}^{{\prime }} (X) = \emptyset$.

The proof of this corollary follows from Eqs. (11), (13), (14) and (15).

#### **Theorem 3**

*Given an information system*$S = (U,A)$, $P \subseteq A{\kern 1pt}$*and*$X \subseteq U$*, the integrated accuracy and the integrated roughness satisfy the relations*$\alpha_{P}^{{\prime }} (X) \le \sigma_{P}^{{\prime }} (X)$*and*$\omega_{P}^{{\prime }} (X) \le \rho_{P}^{{\prime }} (X)$.

It can be concluded from Theorem 3 that the value of the integrated accuracy $\alpha_{P}^{{\prime }} (X)$, which measures two types of uncertainty, will be less than that of $\sigma_{P}^{{\prime }} (X)$, which measures only one type of uncertainty, and the value of the integrated roughness $\rho_{P}^{{\prime }} (X)$, which measures two types of uncertainty, will be greater than that of $\omega_{P}^{{\prime }} (X)$, which measures only one type of uncertainty. Therefore, the new integrated measures $\alpha_{P}^{{\prime }} (X)$ and $\rho_{P}^{{\prime }} (X)$ are logically consistent.

#### **Corollary 4**

*Given an information system*$$ S = (U,A) $$, $$ P,Q \subseteq A $$, $$ P\underset{\raise0.3em\hbox{$\smash{\scriptscriptstyle-}$}}{ \prec } Q $$*and*$$ X \subseteq U $$,

1. *If*$X$*is a boundary rough set (i.e.,*$\underline{{R^{P} }} (X) = \underline{{R^{Q} }} (X) = \emptyset$) *and*$\overline{{R^{Q} }} (X) = \overline{{R^{P} }} (X)$*, then*$\rho_{Q} (X) = \rho_{P} (X)$*and*$\omega_{Q}^{{\prime }} (X) = \omega_{P}^{{\prime }} (X)$*, but*$\rho_{P}^{{\prime }} (X) \le \rho_{Q}^{{\prime }} (X)$;
2. *If*$\rho_{P}^{{\prime }} (X) = \rho_{Q}^{{\prime }} (X)$*, then*$\rho_{Q} (X) = \rho_{P} (X)$*and*$\omega_{Q}^{{\prime }} (X) = \omega_{P}^{{\prime }} (X)$;
3. *If*$\rho_{P} (X) < \rho_{Q} (X)$*or*$\omega_{P}^{{\prime }} (X) < \omega_{Q}^{{\prime }} (X)$*, then*$\rho_{P}^{{\prime }} (X) \le \rho_{Q}^{{\prime }} (X)$;

*Property (1) in Corollary 4 indicates that the integrated roughness*$\rho_{P}^{{\prime }} (X)$*measures both set uncertainty and knowledge uncertainty for*$X$*; however,*$\rho_{P} (X)$*and*$\omega_{P}^{{\prime }} (X)$*measure only set uncertainty. Property (2) in Corollary 4 shows that*$\rho_{P} (X)$*and*$\omega_{P}^{{\prime }} (X)$*are invariant if the integrated roughness*$\rho_{P}^{{\prime }} (X)$*remains unchanged, although the classification granularity is smaller through finer classification. However,*$\rho_{P} (X)$*and*$\omega_{P}^{{\prime }} (X)$*may not decrease if the integrated roughness*$\rho_{P}^{{\prime }} (X)$*decreases. Property (3) in Corollary 4 shows that the integrated roughness*$\rho_{P}^{{\prime }} (X)$*decreases when*$\rho_{P} (X)$*and*$\omega_{P}^{{\prime }} (X)$*decrease. The converses of properties (2) and (3) are not always true. Corollary 4 implies that the integrated roughness is more sensitive than*$\rho_{P} (X)$*and*$\omega_{P}^{{\prime }} (X)$*for a general binary relation.*

The preceding properties characterise the variation of the integrated roughness with the classification granularity. The effectiveness of the proposed measure is verified in the following example.

#### *Example 3* (Continued from Example 1)

Results for the uncertainty measures based on an equivalence relation were obtained from Eqs. (11), (13), (14) and (15), and these results are listed in Table 4.

**Table 4:** New uncertainty measures of a rough set *X* with various classification granularities

	*Num_L*	*Num_U*	*Num_B*	$\sigma_{P}^{{\prime }} (X)$	$\omega_{P}^{{\prime }} (X)$	$H^{{\prime }} (P)$	$G^{{\prime }} (P)$	$\alpha_{P}^{{\prime }} (X)$	$\rho_{P}^{{\prime }} (X)$
(1)	0	3600	3600	0.5	0.5	0.889	0.111	0.445	0.555
(2)	0	2200	2200	0.694	0.306	0.955	0.045	0.663	0.337
(3)	0	2200	2200	0.694	0.306	0.974	0.026	0.676	0.324
(4)	200	2200	2000	0.722	0.278	0.985	0.015	0.711	0.289
(5)	5400	1548	1008	0.860	0.140	0.997	0.003	0.857	0.143
(6)	5400	1548	1008	0.860	0.140	0.997	0.003	0.857	0.143
(7)	5400	1548	1008	0.860	0.140	0.997	0.003	0.857	0.143

From Table 4, we can make the following observations:

1. Comparing partitions (1) with (2), (3) with (4) and (4) with (5), we can observe that the boundary region becomes smaller, and thus $\sigma_{P}^{{\prime }} (X)$ becomes smaller and $\omega_{P}^{{\prime }} (X)$ becomes larger. In addition, the classification granularity in the boundary region becomes finer, which leads to an increase in the discernibility of objects in the boundary region, and thus $\rho_{P}^{{\prime }} (X)$ becomes smaller and $\alpha_{P}^{{\prime }} (X)$ becomes larger. Obviously, the new integrated measures $\alpha_{P}^{{\prime }} (X)$ and $\rho_{P}^{{\prime }} (X)$ reflect not only the set uncertainty but also the knowledge uncertainty in the boundary region.
2. Comparing partition (2) with (3), it can be observed that the boundary region, the global accuracy $\sigma_{P}^{{\prime }} (X)$ and the global roughness $\omega_{P}^{{\prime }} (X)$ do not change. However, the classification granularity in the boundary region becomes finer, i.e., the discernibility of objects in the boundary region increases, and thus $H^{{\prime }} (P)$ becomes larger. Obviously, an increase in $\alpha_{P}^{{\prime }} (X)$ and a decrease in $\rho_{P}^{{\prime }} (X)$ in this case reflect the decrease of the knowledge uncertainty in the boundary region, whereas the set uncertainty does not change.
3. Comparing partitions (5) with (6) and (6) with (7), it can be observed that the boundary region and the classification granularity in the boundary region remain the same, and thus the uncertainty of the rough set *X* does not change. Accordingly, $\sigma_{P}^{{\prime }} (X)$, $\omega_{P}^{{\prime }} (X)$, $H^{{\prime }} (P)$, $G^{{\prime }} (P)$, $\rho_{P}^{{\prime }} (X)$ and $\alpha_{P}^{{\prime }} (X)$ all do not change, which shows that the new integrated measures are unassociated with subdivision of classification granularities unrelated to rough set *X*. Therefore, the new integrated measures are consistent with human cognition.
4. The integrated accuracy $\alpha_{P}^{{\prime }} (X)$ and the integrated roughness $\rho_{P}^{{\prime }} (X)$ reflect two types of uncertainty. Therefore, the value of the integrated accuracy is smaller than those of $\sigma_{P}^{{\prime }} (X)$ and $H^{{\prime }} (P)$, and the value of the integrated roughness $\rho_{P}^{{\prime }} (X)$ is larger than those of $\omega_{P}^{{\prime }} (X)$ and $G^{{\prime }} (P)$. These results are logically consistent.

Example 3 illustrates that the new integrated measures $\alpha_{P}^{{\prime }} (X)$ and $\rho_{P}^{{\prime }} (X)$ for a complete information system overcome the limitations of the existing uncertainty measures, better characterise the imprecision of rough sets and are consistent with human cognition.

#### *Example 4* (Continued from Example 2)

**Fig. 4:**
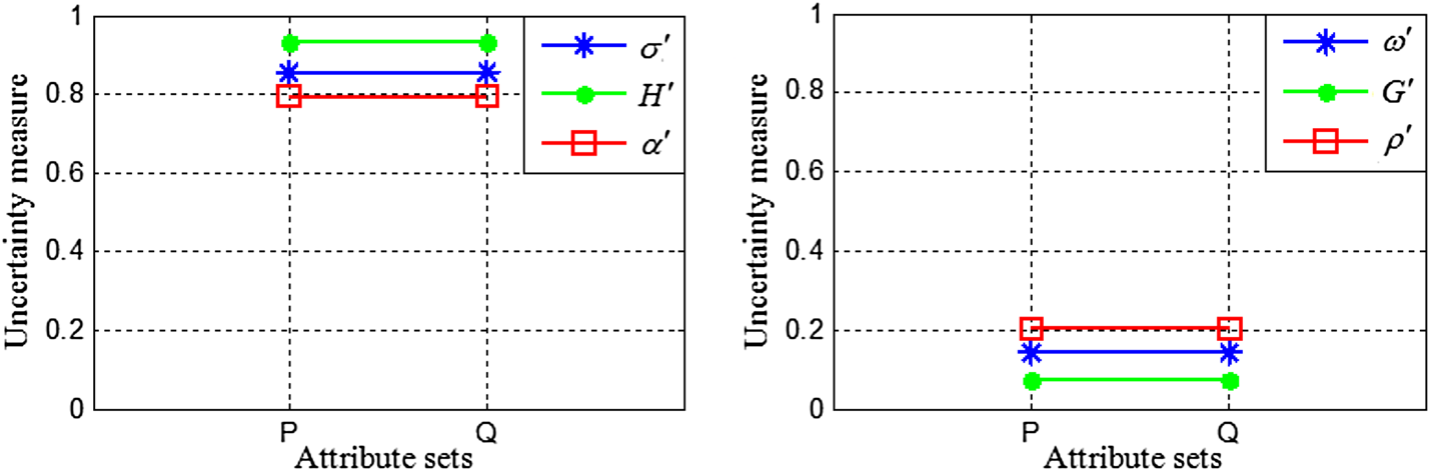
The proposed uncertainty measures when *X* = *X*_1_

**Fig. 5:**
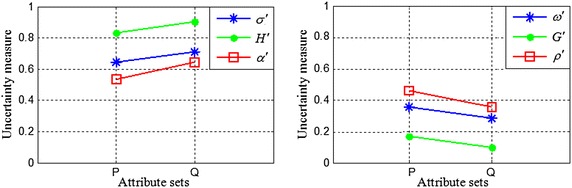
The proposed uncertainty measures when *X* = *X*_2_

We calculate the new uncertainty measures for the tolerance relation using Eqs. (11), (13), (14) and (15). The results are shown in Table 5. Figures 4 and 5 present the new uncertainty measures for $X_{1}$ and $X_{2}$, respectively. The subscripts of the uncertainty measures in Figs. 4 and 5 are omitted (as in Figs. 2 and 3).

**Table 5:** The proposed uncertainty measures for an incomplete information system

*X*	$\sigma_{P}^{{\prime }} (X)$	$\sigma_{Q}^{{\prime }} (X)$	$H^{{\prime }} (P)$	$H^{{\prime }} (Q)$	$\alpha_{P}^{{\prime }} (X)$	$\alpha_{Q}^{{\prime }} (X)$
*X*_1_	0.857	0.857	0.929	0.929	0.796	0.796
*X*_2_	0.643	0.714	0.833	0.905	0.536	0.646

We can draw the following conclusions from Table 5, Fig. 4 and Fig. 5:

1. When $X = X_{1}$, the upper and lower approximations of set $X_{1}$ are equal, and the classification granularities of objects in the boundary region are also the same with respect to the knowledge *P* and *Q*. Thus, subdividing the classification granularities $R_{S}^{P} (u_{3} )$ and $R_{S}^{P} (u_{5} )$ (which are unrelated to *X*) in the negative region of set *X* does not alter the values of $\alpha_{P}^{{\prime }} (X)$ and $\rho_{P}^{{\prime }} (X)$, which shows that $\alpha_{P}^{{\prime }} (X)$ and $\rho_{P}^{{\prime }} (X)$ are consistent with human cognition.
2. When $X = X_{2}$, *X* is a boundary rough set. The boundary regions of *X* with respect to the knowledge *P* and *Q* are different. Consequently, $\sigma_{P}^{{\prime }} (X) < \sigma_{Q}^{{\prime }} (X)$ and $\omega^{\prime}_{Q} (X) < \omega^{\prime}_{P} (X)$. In addition, the classification granularities of objects in the boundary region with respect to the knowledge *P* and *Q* are different. Furthermore, $H^{{\prime }} (P) < H^{{\prime }} (Q)$ and $G^{{\prime }} (Q) < G^{{\prime }} (P)$. Finally, the integrated measures satisfy $\rho_{Q}^{{\prime }} (X) < \rho_{P}^{{\prime }} (X)$ and $\alpha_{P}^{{\prime }} (X) < \alpha_{Q}^{{\prime }} (X)$. Obviously, the proposed integrated accuracy and integrated roughness can not only correctly reflect set uncertainty but also correctly measure knowledge uncertainty for a boundary rough set. Therefore, $\alpha_{P}^{{\prime }} (X)$ and $\rho_{P}^{{\prime }} (X)$ can adequately characterise the uncertainty of rough sets.
3. From Figs. 4 and 5, it can be observed that $\alpha_{P}^{{\prime }} (X) \le \sigma_{P}^{{\prime }} (X)$, $\alpha_{P}^{{\prime }} (X) \le H^{{\prime }} (P)$, $\alpha_{Q}^{{\prime }} (X) \le \sigma_{Q}^{{\prime }} (X)$ and $\alpha_{Q}^{{\prime }} (X) \le H^{{\prime }} (Q)$ when $X = X_{1}$ or $X = X_{2}$. That is to say, the value of the integrated accuracy, which is based on two types of uncertainty, is smaller than that of the measure based on only one type of uncertainty. In addition, $\omega_{P}^{{\prime }} (X) \le \rho_{P}^{{\prime }} (X)$, $G^{{\prime }} (P) \le \rho_{P}^{{\prime }} (X)$, $\omega_{Q}^{{\prime }} (X) \le \rho_{Q}^{{\prime }} (X)$ and $G^{{\prime }} (Q) \le \rho_{Q}^{{\prime }} (X)$, which indicates that the value of the integrated roughness, which reflects two types of uncertainty, is greater than that of the measure reflecting only one type of uncertainty. Obviously, these results are logically consistent.

Comparing Examples 3 and 4 with Examples 1 and 2, we can conclude that the new integrated measures $\alpha_{P}^{{\prime }} (X)$ and $\rho_{P}^{{\prime }} (X)$ under general binary relations are suitable for both complete and incomplete information systems. These new measures overcome the limitations of existing uncertainty measures and can satisfactorily characterise the imprecision of rough sets. Therefore, the proposed integrated measures are more comprehensive and effective uncertainty measures for both complete and incomplete information systems.

## Conclusion

The extension of RST to incomplete information systems is important for making RST practical. Uncertainty measures are the basis for information processing and knowledge acquisition in an incomplete information system. At present, direct processing of an incomplete information system lacks a theoretical basis. By considering the nature of the roughness of sets, we developed new integrated measures based on general binary relations. Several desirable properties of the proposed measures have been shown. We have demonstrated that the new measures overcome the limitations of existing uncertainty measures and can be used to measure with a simple and comprehensive form the roughness and the accuracy of a rough set, and the results are logically consistent. Research on the application of our proposed integrated measures for rule acquisition is planned.
